# Antagonism of Some Aquatic Hyphomycetes against Plant Pathogenic Fungi

**DOI:** 10.1100/tsw.2010.80

**Published:** 2010-05-04

**Authors:** S. C. Sati, P. Arya

**Affiliations:** Department of Botany, Kumaun University, Nainital, India

**Keywords:** antagonism, aquatic hyphomycetes, root endophytic fungi, Tetrachaetum elegans, Heliscus lugdunensis

## Abstract

The antagonistic activity of five aquatic hyphomycetes, viz., *Heliscus lugdunensis*, *Tetrachaetum elegans*, *Tetracladium breve*, *T. marchalianum*, and *T. nainitalense*, against seven plant pathogenic fungi was studied using a dual culture technique. Inhibitory activity of tested aquatic hyphomycetes was determined by measuring the radial growth of plant pathogenic fungi on dual culture plates. *Tetrachaetum elegans* showed antagonistic activity against *Colletotrichum falcatum*, *Fusarium oxysporum*, *Pyricularia oryzae*, *Sclerotium sclerotiorum*, and *Tilletia indicaHeliscus lugdunensis* showed antagonism against only two plant pathogenic fungi, *Rhizoctonia solani* and *Colletotrichum falcatum*.*Tetracladium breve*, *T. marchalianum*, and *T. nainitalense* showed no response towards tested plant pathogenic fungi.

## INTRODUCTION

Aquatic hyphomycetes are conidial fungi that are abundant in almost all freshwater bodies throughout the world[1,2,3]. Occurrence of these fungi as root endophytes in healthy plants indicates that they are biologically important and may have a beneficial role in plant health[4,5]. The intra- and interspecific interaction of aquatic hyphomycetes in relation to aquatic ascomycetes and release of diffusible inhibitory substances have been reported by some workers[6,7,8].

Interaction of aquatic hyphomycetes with bacteria and terrestrial fungi has been suggested by Chamier et al.[9] and Gulis and Suberkropp[10]. A new antimicrobial compound "Quinapathin" has been described from the aeroaquatic hyphomycete *Helicoon richonis*[11,12]. Likewise, isolation and structural determination of the antimicrobial compound "Anguillosporal", from *Anguillospora longissima*, has resulted in the discovery of a new metabolite[13].

Previously, Platas et al.[14] and Gulis and Stephanovich[15] demonstrated the antagonistic activity of a few aquatic hyphomycetes. According to Gloer[16], the secondary metabolites of aquatic hyphomycetes could result in the discovery of new natural bioactive products of medicinal and agricultural importance. In a study on antibmicrobial effects of aquatic hyphomycetes, Gulis and Stepahnovich[15] suggested that due to their specific habitat, they may have biosynthetic capabilities different from those of terrestrial fungi.

A number of aquatic hyphomycetes have been isolated as root endophytes of some plants[17,18,19]. Nevertheless, the information on the antagonistic activity of aquatic hyphomycetes is quite meager. The present investigation evaluated the antagonistic activity of *Heliscus lugdunensis* Sacc. & Therry, *Tetrachaetum elegans* Ingold, *Tetracladium breve* Roldan, *T. marchalianum* De Wildeman, and *T. nainitalense* Sati & Arya isolated from the roots of riparian plants against plant pathogenic fungi.

## MATERIALS AND METHODS

Roots of healthy riparian plants (Table 1) were collected from ravine areas near Nainital, Uttarakhand, India (29.39º N 79.45º E) in the western part of Central Himalaya. Root pieces (1–2 cm in size) were washed in running water, then surface sterilized by immersing them in a 0.01% sodium hypochlorite solution for 5 min and then in 96% ethanol for 30 sec[17]. The pieces were placed onto 2% Malt Extract (ME) agar and incubated at 20 ± 2°C for 10–15 days in the dark. Isolations were made from the hyphal growth emanating from the root pieces. The isolates were identified with the help of relevant monographs and papers (Table 1).

**TABLE 1**
**Fungal Isolates, Their Host, and Location at Nainital, India**

| Fungal Isolates | Host Plant | Location |
|---|---|---|
| *Tetrachaetum elegans* Ingold | *Pilea scripta* (Buch.-Ham. Ex D. Don) Wedd. | Vinayak |
| *Heliscus lugdunensis* Sacc. & Therry | *Strobilanthes alatus* Nees | Ratighat |
| *Tetracladium marchalianum* De Wildeman | *Geranium nepalense* Sweet | Vinayak |
| *T. breve* Roldan | *Eupatorium adenophorum* Sprengel | Gufa Mahadev |
| *T. nainitalense* Sati & Arya | *E. adenophorum* Sprengel | Gufa Mahadev |

Seven plant pathogenic fungi (test fungi), viz., *Colletotrichum falcatum*, *Fusarium oxysporum*, *Pyricularia oryzae*, *Rhizoctonia solani*, *Sclerotinia sclerotiorum*, *Sclerotium rolfsii*, and *Tilletia indica* were obtained from G.B. Pant University of Agriculture and Technology, Pantnagar, (USN), India.

## Antagonistic Activity (Dual Culture)

Antagonistic activity of aquatic hyphomycetes against the test fungi was studied using a dual culture technique. Mycelial disks (5 mm diameter) of aquatic hyphomycetes and the test fungi were cut from the periphery of actively growing colonies and aseptically placed 2.5 cm apart on assay plates (90 mm diameter) containing 15 ml of 2% ME agar (Figs. 1 and 2). Three replicate plates of each combination of dual cultures were incubated at 20 ± 2°C for 4 days in the dark.

The antagonistic activity of aquatic hyphomycetes was analyzed by measuring the radial growth of the test fungi on day 4 following inoculation. The measurements were taken in two directions: $R_1$ (radius in opposite direction) and $R_2$ (radius in direction of the aquatic hyphomycetes). $R_1$ distance was also considered as negative control. Percent inhibition of radial growth was calculated as suggested by Fokkema[20] and Shearer and Zare-Maivan[8]:

$$\text{Percent Inhibition} = \frac{R_1 - R_2}{R_1} \times 100$$

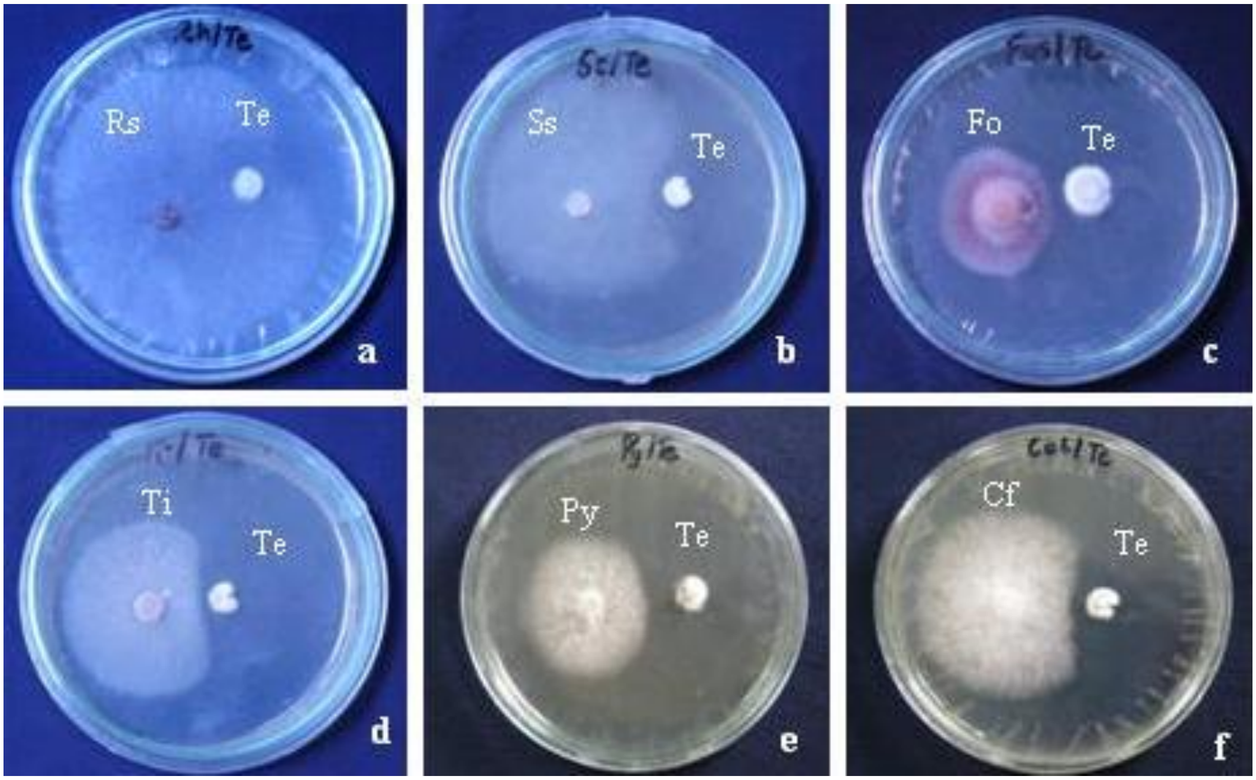

**FIGURE 1.** Inhibition of plant pathogenic fungi by *Tetrachaetum elegans*: (a) *Rhizoctonia solani*, (b) *Sclerotinia sclerotiorum*, (c) *Fusarium oxysporum*, (d) *Tilletia indica*, (e) *Pyricularia oryzae*, (f) *Colletotrichum falcatum*.

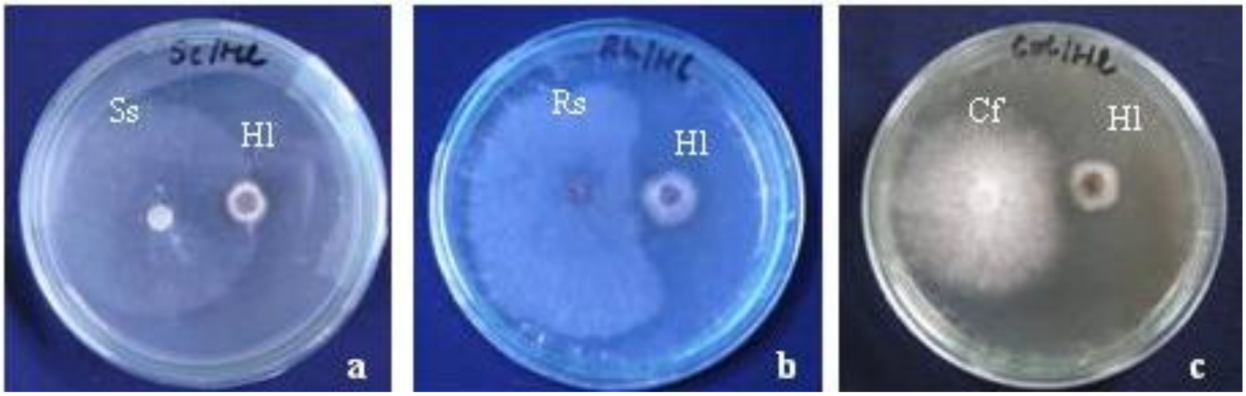

**FIGURE 2.** Inhibition of plant pathogenic fungi by *Heliscus lugdunensis*: (a) *Sclerotinia sclerotiorum*, (b) *Rhizoctonia solani*, (c) *Colletotrichum falcatum*.

## Analysis of Data

The differences in radial length were analyzed statistically using paired t-tests. A mean value of percent inhibition followed by standard error of mean (SEM) was also determined.

## RESULTS AND DISCUSSION

The antagonistic activity of five aquatic hyphomycetes against seven plant pathogenic fungi is presented in Table 2. *Tetrachaetum elegans* showed a significant percent ($p < 0.05$) of inhibitory activity towards five plant pathogenic fungi, viz., *Colletotrichum falcatum*, *Fusarium oxysporum*, *Pyricularia oryzae*, *Sclerotinia sclerotiorum*, and *Tilletia indica* (Fig. 1, b–f). *Heliscus lugdunensis* showed inhibitory activity, but only towards two plant pathogenic fungi, viz., *Rhizoctonia solani* and *Colletotrichum falcatum* (Fig. 2, b–c). *Tetracladium marchalianum*, *T. breve*, and *T. nainitalense* exhibited no inhibitory effect against the tested plant pathogenic fungi (Table 2).

**TABLE 2**
**Percent Inhibition of Seven Pathogenic Fungi Against Five Aquatic Hyphomycetes (± SEM Based on Three Replicates)**

| | **Test Fungi** | **Fungal Isolates** | | | | |
|---|---|---|---|---|---|---|
| | | ***Tetrachaetum elegans*** | ***Heliscus lugdunensis*** | ***Tetracladium marchalianum*** | ***T. breve*** | ***T. nainitalense*** |
| % Inhibition of Test Fungi | *Rhizoctonia solani* | — | 45.24 ± 0.03 | — | — | — |
| | *Pyricularia oryzae* | 27.05 ± 0.15 | — | — | — | — |
| | *Sclerotinia sclerotiorum* | 25.63 ± 0.06 | — | — | — | — |
| | *Colletotrichum falcatum* | 33.64 ± 0.10 | 25.28 ± 0.06 | — | — | — |
| | *Fusarium oxysporum* | 30.89 ± 0.06 | — | — | — | — |
| | *Sclerotium rolfsii* | — | — | — | — | — |
| | *Tilletia indica* | 30.42 ± 0.06 | — | — | — | — |

— = No activity.

The paired t-test performed on radial growth values of *Tetrachaetum elegans* were significantly different for *Pyricularia oryzae* ($p < 0.05$), *Sclerotinia sclerotiorum* ($p < 0.05$), *Colletotrichum falcatum* ($p < 0.05$), *Fusarium oxysporum* ($p < 0.01$), and *Tilletia indica* ($p < 0.01$). *Heliscus lugdunensis* differences were significant for *Rhizoctonia solani* ($p < 0.01$) and *Colletotrichum falcatum* ($p < 0.01$).

In the present study, endophytic aquatic hyphomycetes isolated from riparian plant roots were screened for their antifungal activity against seven plant pathogenic fungi. Earlier antagonistic studies using metabolites of ectomycorrhizal fungi and other endophytic fungi showed positive results[15,21,22,23]. However, in this study, the antagonistic activity of aquatic hyphomycetes against plant pathogenic fungi was tested by using a dual culture technique (Figs. 1 and 2). It was interesting to note that *H. lugdunensis* showed a significant antagonistic activity against *R. solani* and *C. falcatum* (Fig. 2), whereas Gulis and Stephanovich[15] observed *H. lugdunensis* as biologically inactive against Gram-negative and Gram-positive bacteria, yeast, and hyphomycetes when using metabolites in an “agar well” technique. The variation in the present study may be due, perhaps, to the use of different techniques. This may also be supported by the observation of Tian et al.[24] that endophytic fungi showed antagonism in the dual culture test, whereas their metabolites showed no or little activity.

All the previous studies on antifungal activity were determined by using metabolites of endophytic aquatic hyphomycetous fungi, but the present investigation was conducted to check the antagonistic role of these fungi by using dual culture for the first time. The present study clearly indicates that aquatic hyphomycetous fungi synthesize substances that inhibit the growth of certain phytopathogens. However, further screening of the compounds responsible for the antagonistic activity is required. This antagonistic property of aquatic hyphomycetes may also be used in the future for the exploitation of naturally occurring compounds, which can compete with synthetic fungicides.

## ACKNOWLEDGMENTS

This study was supported by a grant from the Department of Science and Technology (DST), Government of India. The authors would like to thank the Head of the Department of Botany, Kumaun University, Nainital, India for providing the lab facility and Dr. Kapil Khulbe for his help in preparing the experiment. The paper benefited from the suggestions of three anonymous reviewers and the editors.
